# A Rare Presentation of a True Intra-articular Lipoma: A Case Report and Review of Imaging Findings

**DOI:** 10.7759/cureus.25094

**Published:** 2022-05-18

**Authors:** Areez Shafqat, Shameel Shafqat, Taha M Ahmed, Tarek Z Arabi, Belal N Sabbah, Jibran A Khan, Wael K Alfehaid, Syed S Islam

**Affiliations:** 1 College of Medicine, Alfaisal University College of Medicine, Riyadh, SAU; 2 College of Medicine, Aga Khan University Medical College, Karachi, PAK; 3 Radiology, King Salman Hospital, Riyadh, SAU

**Keywords:** diagnostic imaging, musculoskeletal radiology, lipoma arborescens, knee joint, lipoma

## Abstract

This report describes the unique case of an intra-articular lipoma in a 34-year-old male. The patient presented with a history of chronic knee pain associated with an intermittent sensation of the knee giving way. Physical examination and initial radiographic imaging were unremarkable. Magnetic resonance imaging (MRI) revealed a 9.2 x 6.7 mm ovoid mass posterior to the posterior cruciate ligament (PCL) exhibiting hyperintense signals on T1-weighted images and intermediate-to-high intensity signals on T2. On subsequent proton density fat suppression sequences, the mass demonstrated homogenous signal suppression and was confirmed as being a lipoma. To the best of our knowledge, this is only the second reported case of an intra-articular lipoma arising posterior to the PCL. Intra-articular lipomas, albeit rare, should be considered in the differential diagnosis for chronic knee pain with associated joint motion abnormalities. MRI remains the gold standard in imaging intra-articular soft tissue pathology and should be the study of choice in differentiating intra-articular lipomas from similar conditions such as pigmented villonodular synovitis and lipoma arborescens.

## Introduction

Lipomas, benign tumors of fat, are common soft tissue tumors. They commonly affect middle-aged individuals between 40 and 60 and usually occur just under the skin [1]. Deeper origins are uncommon in comparison. Intra-articular lipomas are extremely rare; a review by Kheok et al. in 2017 reported a total of 27 cases, 19 of which were in the knee [2].

True intra-articular lipomas are solitary round or ovoid masses of fat contained within a fibrous capsule. True intra-articular lipomas most often occur in the knee joint, commonly in the anterior compartment on Hoffa’s fat pad or the suprapatellar pouch, although they occasionally affect the hip, spine, shoulders, elbows, and wrists [3]. Rarely do they reside in the posterior compartment behind the posterior cruciate ligament (PCL). From our literature search, we found one such case [4]. It is crucial to distinguish true intra-articular lipomas from lipoma arborescens for effective management. Therefore, the investigating radiologist requires a thorough understanding of the imaging features of different intra-articular masses. Therefore, the purpose of this article is to familiarize providers with these differential imaging features.

We report a 34-year-old male with a true intra-articular lipoma posterior to the PCL. Through a literature review, we discuss the appropriate differentials and imaging features that radiologists must be acquainted with.

## Case presentation

A 34-year-old male, otherwise healthy, presented complaining of chronic right knee pain and a feeling of the knee giving way. Meniscal degeneration or tear was suspected, and the patient was referred to us for radiological evaluation.

**Figure 1:**
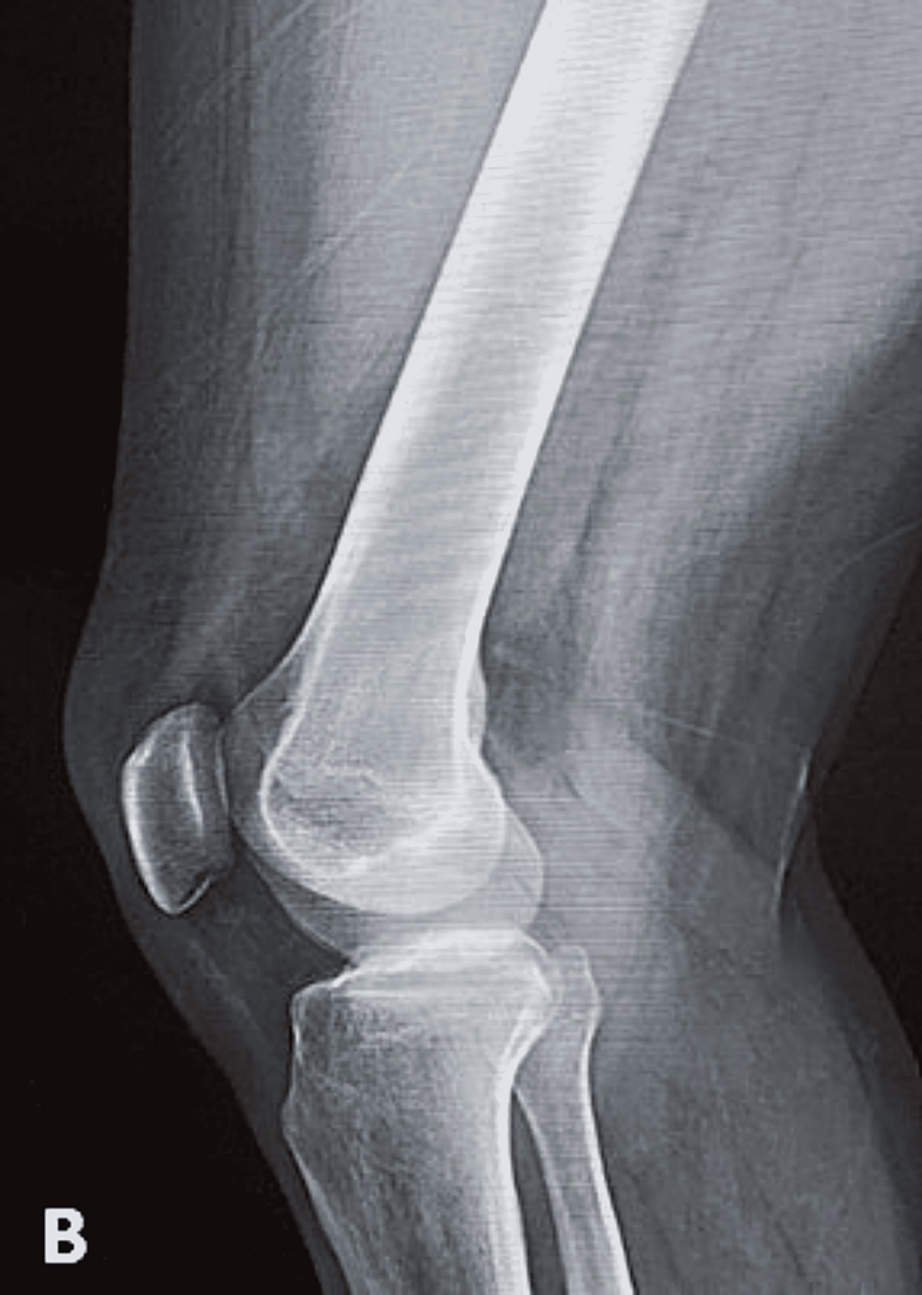
Normal (B) lateral view X-ray of the knee showing no soft-tissue swelling or osteodegenerative changes

**Figure 2:**
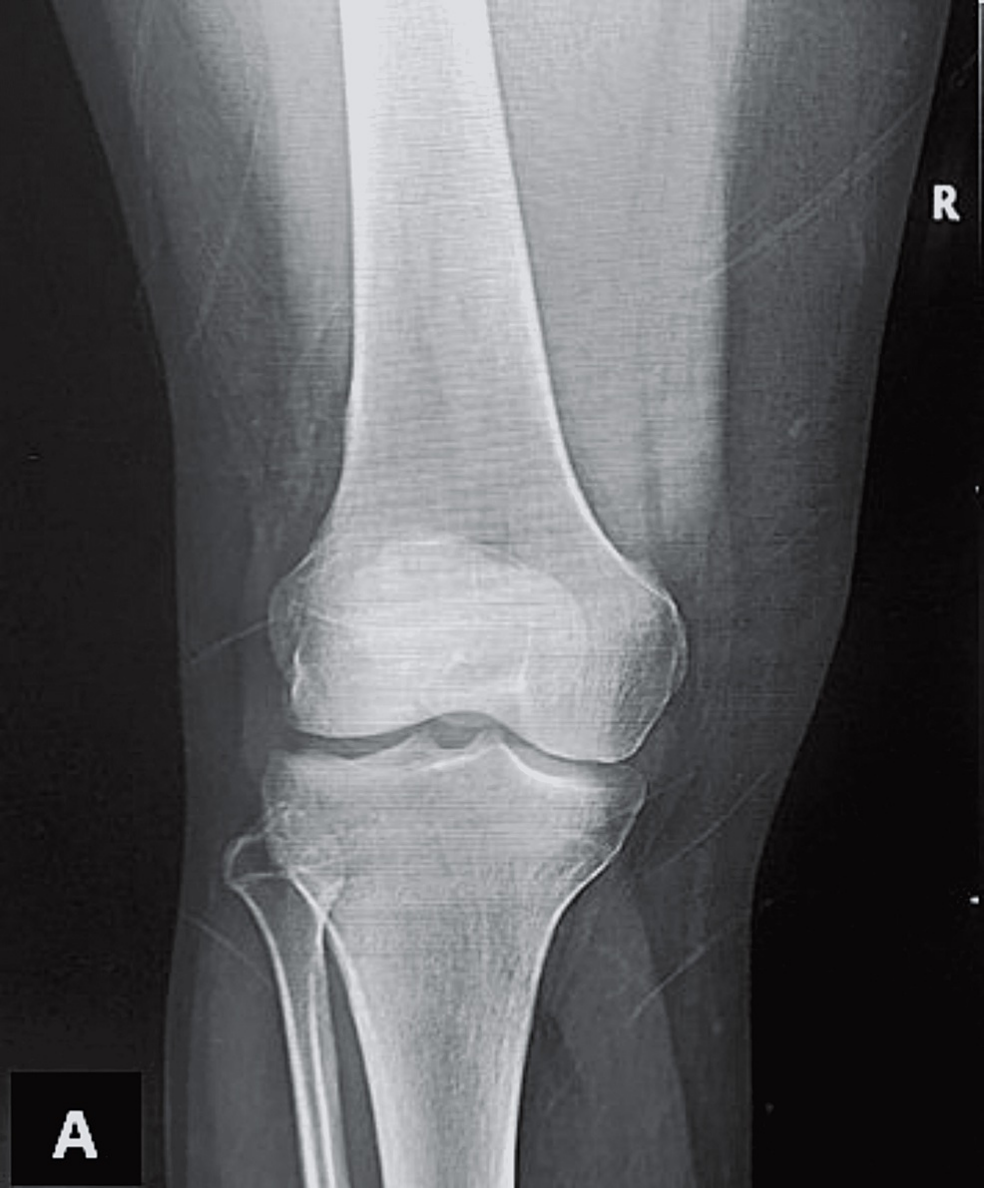
Normal A/P view X-ray of the knee showing no soft-tissue swelling or osteodegenerative changes A/P: anteroposterior

The initial X-ray of the right knee was normal and revealed no soft tissue swelling or degenerative changes (Figures 1-2).

**Figure 3:**
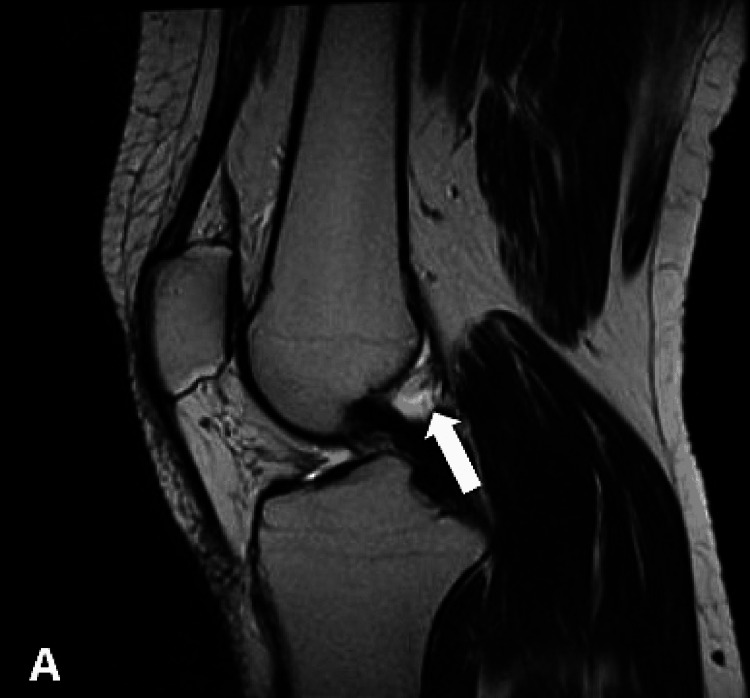
Sagittal T2 MRI showing a small ovoid hyperintense intra-articular lipoma adjacent to PCL (arrow) PCL: posterior cruciate ligament

**Figure 4:**
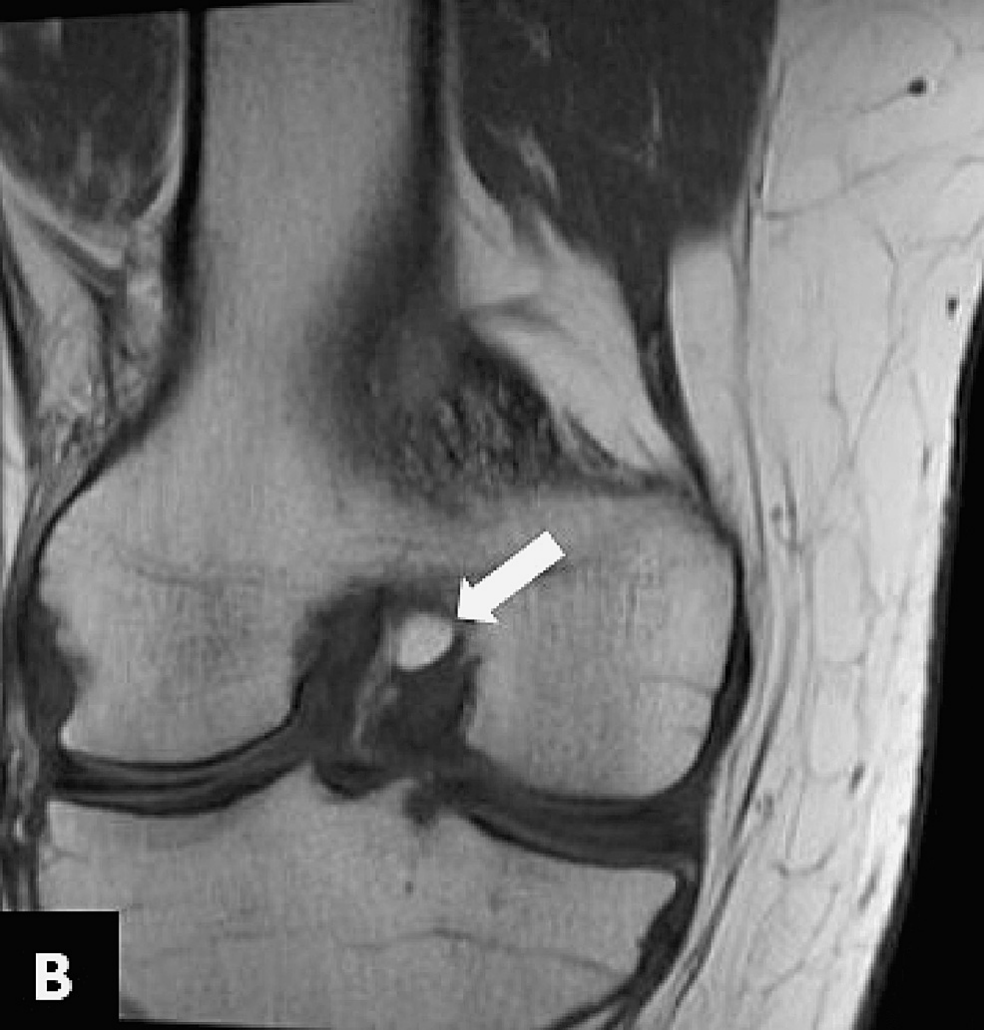
Coronal T1 MRI demonstrating an intra-articular lesion exhibiting fat signals posterior to the PCL (arrow) PCL: posterior cruciate ligament

**Figure 5:**
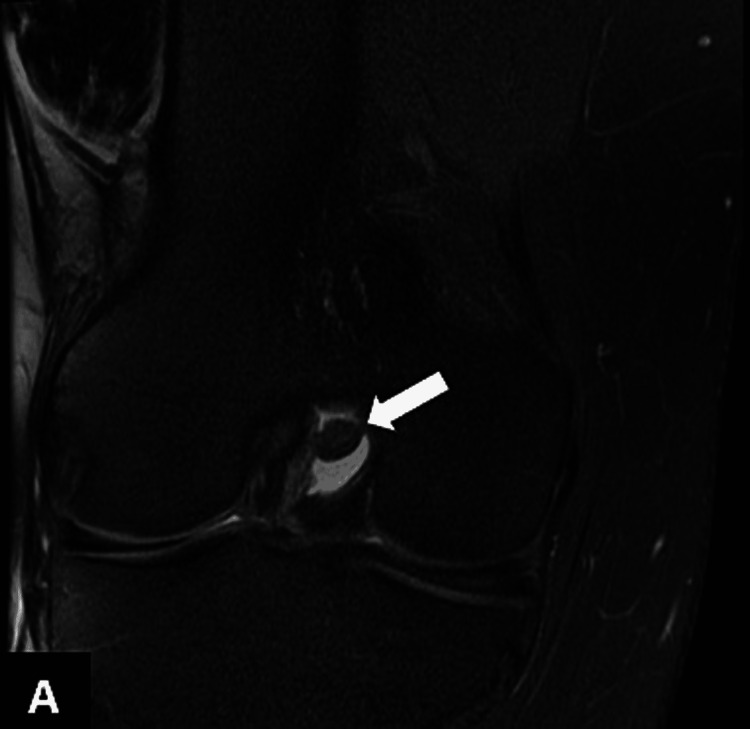
Coronal PDFS sequences revealing suppression of intra-articular signals suggestive of lipoma PDFS: proton density fat suppression

**Figure 6:**
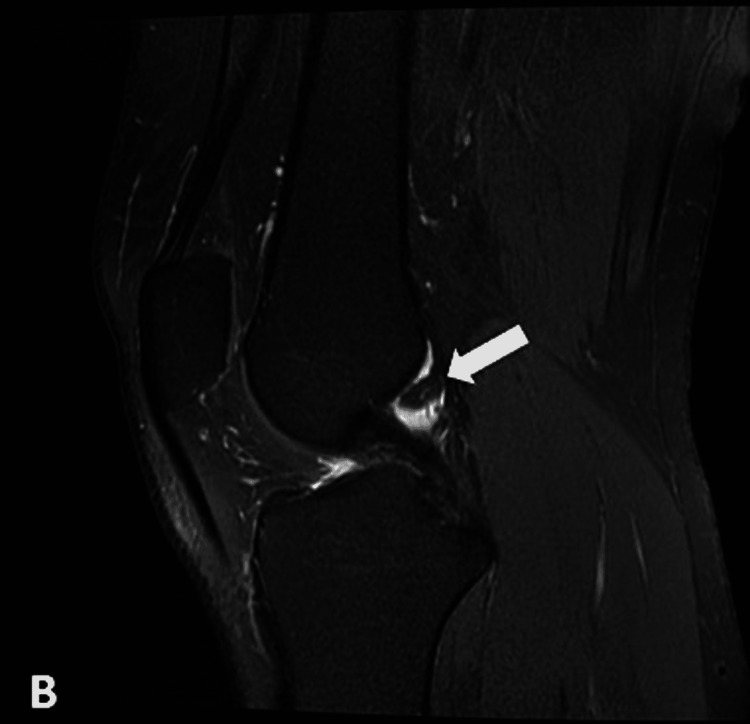
Sagittal PDFS sequences revealing suppression of intra-articular signals suggestive of lipoma PDFS: proton density fat suppression

Subsequent magnetic resonance imaging (MRI) of the right knee demonstrated a small ovoid area of abnormal signal intensity was noted posterior to the PCL. It measured 9.2 x 6.7 mm and exhibited hyperintense signals on T1-weighted images and intermediate-to-high intensity signals on T2 (Figures 3-4) and signals were suppressed on proton density fat suppression (PDFS) sequences (Figures 5-6).

Minimal associated effusion was observed in the tibiofemoral joint, mild edematous changes were seen in the prepatellar soft tissues, and no additional findings were noted. A diagnosis of small intra-articular lipoma posterior to the PCL was made on this basis. Unfortunately, we could not procure a biopsy specimen for histopathologic confirmation of lipoma, but the signal suppression on PDFS sequences confirms a lipomatous nature, and the imaging evaluation strongly suggested probable intra-articular lipoma.

We managed the patient symptomatically and advised him for regular follow-ups because we lack an arthroscopic facility at our hospital.

## Discussion

Intra-articular lipomas are exceedingly rare. They are space-occupying lesions and present clinically with symptoms of mass effect, most commonly pain and limited range of movement. They can alternatively manifest with locking of the knee [5-6] - by getting impinged between various joint structures - and snapping of the patellofemoral joint due to loose bodies within the subsynovium [7]. Intra-articular lipomas can also be asymptomatic, only coming to medical attention on routine medical checkups [8] or becoming painful upon getting inflamed secondary to knee trauma [9]. The only other reported case of intra-articular lipoma in the posterior knee compartment reported a 51-year-old woman with intermittent left knee pain, especially during flexion [4], which is consistent with our case, further highlighting the importance radiology plays in the diagnostic workup of these clinically non-specific lesions.

The role of plain X-ray in the diagnosis of intra-articular lipoma is limited. Small lipomas may not be detected, whereas larger lesions may feature isolated soft-tissue swelling with no other joint changes [10-11]. In the present case, the X-ray was normal, and the previous report on posterior compartment intra-articular lipoma showed only mild degenerative changes on radiography, which may be unrelated to the lipoma, as their patient was considerably older than ours [4].

MRI is the diagnostic tool of choice because it offers a more soft tissue definition than other modalities such as computed tomography (CT). Intra-articular lipomas exhibit high-intensity signals on T1- and T2-weighted images, which suppress fat suppression sequences [11]. However, non-specific MRI findings are also possible, exhibiting fluid-like signal intensity owing to mucoid degeneration. Other reports also use CT and report low-attenuation between -65 and -120 Hounsfield Units (HU), suggestive of a lipomatous consistency [4]. We only used MRI for diagnosing this lesion, limiting our ability to comment on imaging findings of intra-articular lipomas on other imaging modalities.

Sheldon et al. classified the differential diagnosis of intra-articular mass lesions based on underlying pathogenesis [12]. The most important differential is lipoma arborescens, which is characterized by villous proliferation of subsynovial adipose tissue [13]. Lipoma arborescens most commonly occurs between the fifth to seventh decades of life and is a feature of degenerative joint disease. These lesions typically involve the knee and manifest as painless joint swelling, sometimes causing pain or locking of the knee [13]. On radiographs, lipoma arborescence can manifest with joint effusions and associated osteoarthritis, but space-occupying lesions are rarely detected. MRI reveals a frond-like lesion showing high-intensity signals on T1 and T2 [12-13]. Furthermore, ultrasound (US) reveals a frond-like hyperechoic mass and associated joint effusion. When viewed in real-time, the mass moves wave-like on compression and manipulation [12-13]. Confirming lipoma arborescens is based on macroscopic appearance after resection [12]. The gross appearance of lipoma arborescens confirms radiologic findings, showing a frond-like or finger-like lipomatous proliferation [14].

Pigmented villonodular synovitis (PVNS), a benign but locally aggressive monoarticular synovial disease characterized by villous or nodular synovial expansion and hemosiderin deposition, is another important differential [15]. This lesion affects young-to-middle-age adults and most commonly involves the knee joint. PVNS also may not be detected on radiographs. Therefore, MRI again is crucial for differentiating between PVNS, lipoma arborescens, and intra-articular lipomas [12,15]. PNVS usually manifests characteristically on MRI as diffuse joint involvement and low-intensity signals on all MR sequences [12,15]. Lastly, the presence of hemosiderin - a paramagnetic substance - creates a blooming effect on gradient-echo MR sequences due to a positive magnetic susceptibility [15].

Lastly, peri-cruciate fat pad inflammation is another lipomatous cause of non-specific posterior knee pain, occurring mainly in young, physically active adults, similar to our patient. The peri-cruciate fat pad situates in the intercondylar fossa between the cruciate ligaments. MRI reveals increased signals on PDFS sequences and post-contrast enhancement [3].

A PCL ganglion cyst is another common differential to consider. Like intra-articular lipomas, ganglion cysts are more common in the anterior compartment and present non-specifically with knee pain, limited ROM, and/or snapping and locking of the knee. On imaging, they classically appear as well-defined cystic masses adjacent to the PCL, which display hypointense signals on T1 and T2-weighted sequences and do not suppress on PDFS, differentiating them from lipomas [16].

Lastly, liposarcomas are the second most common soft-tissue sarcomas and can rarely develop in the knee joint. Like intra-articular lipomas, they affect middle-aged individuals. However, they are locally aggressive tumors, manifested clinically as a rapidly growing painless soft-tissue swelling around the knee joint and a requirement for radical amputation if diagnosis and intervention are delayed [17]. Furthermore, they can metastasize to the lungs, which can be fatal. MRI in the setting of liposarcoma reveals a large, locally infiltrative mass - sometimes accompanied by extra-articular spread - exhibiting mostly lipomatous signals but also non-fatty components in the form of thick septations [18], distinguishing them from benign lipomas. However, high-grade liposarcomas lack a lipomatous component and appear similar to other sarcomas. A chest CT must also be performed to assess for pulmonary metastasis [17,19].

In summary, a myriad of conditions - both benign and malignant - can involve the knee joint all of which present similarly on clinical evaluation. Imaging, particularly MRI, is essential in the diagnostic workup of intra-articular masses, but a biopsy is essential when imaging evaluation is uncertain to exclude rare etiologies like intra-articular liposarcoma before surgical intervention. Surgical consultations are curative. Both arthroscopy and arthrotomy are viable treatment options in the literature. However, arthroscopic resection may not be feasible for larger lesions, favoring arthrotomy [11].

## Conclusions

We report the second case of intra-articular lipoma in the knee joint posterior to the PCL. Intra-articular lipomas have a non-specific clinical presentation, making a thorough radiologic evaluation essential. MRI is the imaging modality of choice in diagnosing intra-articular mass lesions while plain radiographs have a relatively limited utility. Key differentials of intra-articular lipoma to rule out include lipoma arborescens and PVNS. Rare etiologies to consider include intra-articular liposarcoma, ganglion cyst, and peri-cruciate fat pad inflammation. When imaging evaluation is uncertain, a biopsy should be done to carry out a histopathological diagnosis. The major limitation of the present study was us being unable to confirm our diagnosis histologically after surgical resection. Nevertheless, the imaging findings were strongly suggestive of lipoma, and we hope that practicing radiologists familiarize themselves with the aforementioned differential imaging findings of these lesions to prevent complications of misdiagnosis.
